# ‘Ethics Between the Lines’ – Nurses’ Experiences of Ethical Challenges in Long-Term Care

**DOI:** 10.1177/23333936211060036

**Published:** 2022-01-05

**Authors:** Hilde Munkeby, Aud Moe, Grete Bratberg, Siri A. Devik

**Affiliations:** 1 Faculty of Nursing and Health Sciences, 158927 Nord University, Levanger, Norway; 2 Centre of Care Research, Steinkjer, Mid-Norway, Faculty of Nursing and Health Sciences, 158928 Nord University, Namsos, Norway

**Keywords:** focus groups, content analysis, ethical challenges, nursing, community healthcare, Norway

## Abstract

In long-term care, ethical challenges are becoming increasingly apparent as the number of older patients with complex care needs increases, in parallel with growing demands for more cost-efficient care. Scarce resources, cross-pressure and value conflicts are associated with missed care, moral stress and nurses wanting to leave the profession. Through five focus group interviews, this study aimed to explore how nurses working in nursing homes and homecare services perceive, experience and manage ethical challenges in everyday work. Content analysis revealed three main themes: striving to do good; failing and being let down and getting rid of frustrations and learning from experiences. The nurses’ morality was mainly expressed through emotions that arose in specific situations. Dedicated spaces for ethical reflection and leaders who recognize that organizational conditions affect the individual nurse-patient relationship are required. Facilitating ethical reflection is an important leadership responsibility, which may also require leaders to actually participate.

## Introduction

In long-term care settings, such as nursing homes and home nursing care, ethical challenges are becoming increasingly apparent as the number of older patients with complex and long-term care needs increases, in parallel with growing demands for more cost-efficient care (Preshaw et al., 2016). Reduction in hospital admissions, early discharge practices and delays in the allocation of institutional space are seen as contributing to increased task pressure in long-term care (Henderson et al., 2017). As front-line workers, nurses have to deal with increased workload and they have a great influence on the quality of care that is actually given (Kalánková et al., 2019), but often find that they are unable to provide the desired level of care. Scarce resources, cross-pressure and value conflicts increase the risk of missed care, moral stress, sick leave and the number of nurses who want to leave the profession (Indregard et al., 2018; Kristiansen et al., 2015; Morley et al., 2020; Tønnessen et al., 2020). Long-term care is typically characterized by long-lasting and close relationships between nurses and patients (McCormack et al., 2017), few colleagues to consult with (Lillemoen & Pedersen, 2013) and concerns about care quality and capacity (Botngård et al., 2021; Holm et al., 2016). Therefore, it is especially important to examine nurses' experience with and handling of daily ethical challenges in this context.

Although most research in this field has been conducted in hospitals (Rainer et al., 2018), issues such as end-of-life care, patients who refuse care, prioritization involving clashes of ethical principles, communication skills and organization resources have been found to be especially ethically demanding in nursing homes and home nursing care (Bollig et al., 2015; Heggestad et al., 2020; Preshaw et al., 2016). Lillemoen and Pedersen (2013) indicate that issues related to scarce resources and lack of knowledge and skills are the most prevalent causes of ethical challenges in these settings. Other researchers point to the great diversity in the patient group and the need for close collaboration with patients and relatives as particularly challenging (Heggestad et al., 2020). Our study is motivated by the lack of knowledge in this area, especially in-depth knowledge obtained from nurses, who are expected to ensure that an increasing number of people receive proper health care outside hospitals (Milliken & Grace, 2017; Rasoal, 2020).

Studies show that nurses are not always aware of the ethical dimension of their work and may overlook ethical issues or even adjust work routines to avoid them (Høy et al., 2016; Jakobsen & Sørlie, 2010; Milliken & Grace, 2017; Svanström et al., 2013). Inadequate handling of ethical issues may not only lead to lower moral sensitivity and quality of care provided, but also put strain on the nurses themselves (Fida et al., 2016; Lamiani et al., 2017).

Although some have called for more attention to ethics as a collective and managerial responsibility (Lützén & Kvist, 2012), dealing with ethics is predominantly regarded as an individual responsibility – specifically, as part of being a professional (Devik et al., 2020; Juthberg et al., 2007; Pauly et al., 2012). In order to strengthen individual healthcare workers’ handling of ethical challenges in long-term care, ethics reflection groups have been established that have proved both meaningful and helpful (Lillemoen & Pedersen, 2013; Magelssen et al., 2018). Nevertheless, making ethics understandable and the subject of reflection may be difficult. Problems with conceptualizing ethics and separating ethical reflection from general reflection in practice have been raised (Karlsen et al., 2019).

Principle-based ethics have played a dominant role in nursing until recently, but both caring ethics and feminist ethics have evolved and shed new light on the ethical aspect in healthcare settings (Gastmans, 2013). In this study, we chose not to let a particular theory or definition of ethical challenges govern. Instead, we wanted to let the nurses' own understandings be heard. We were inspired by Margaret Urban Walker (1998), who notes that ‘morality consists of practices, not theories’, and decided to address ethics from the bottom up, based on the practices of those involved. The aim of this study was therefore to explore how nurses working in long-term care perceive, experience and manage ethical challenges in their everyday work.

## Methods

### Design

Qualitative exploration by the use of focus group discussions were considered suitable for accessing nurses’ descriptions, opinions and reflections about experiences of ethical challenges in their everyday work (Patton, 2002). Focus group interviews especially promote interaction and discussion between participants, which can reveal common knowledge, as well as new and different perspectives that would not otherwise emerge (Kitzinger, 1994). Compared to individual interviews, the group process is beneficial in that it helps people to identify themselves and clarify their views (Kitzinger, 1995).

### Participants and Setting

Healthcare services in Norway are a public responsibility and divided into two levels. Hospital services are under the Ministry of Health and Care Services and are organized in a business model, while municipalities administer and are responsible for nursing homes and home nursing care services.

This study was part of a collaborative project between three municipalities and a research group in Mid-Norway. The project aimed to explore ethical competence among healthcare workers in the municipalities’ nursing homes and home care services. Nursing leaders (first-level leaders) in the municipalities were members of the project group and participated both in the design of the study and the recruitment of participants. The municipalities themselves prioritized which services would be covered by the project. This resulted in nursing homes becoming the context in one municipality, home nursing care becoming the context in the second municipality and both service areas becoming the context in the third municipality.

The recruitment criteria were that the participants worked in nursing home or home care services and had at least 2 years of experience in care work. Recruitment was based on convenience and volunteerism. The nursing leaders who invited and requested their employees to participate sought representation from both care settings and from various professional groups that reflected the skill-mix that normally exists in the services. The leaders’ knowledge of their employees was seen as an advantage when approaching participants who could potentially actively contribute in the group discussions.

The wards and units that were invited covered general somatic care, dementia care, geriatric psychiatry, rehabilitation and acute care. After giving written consent, the participants were organized into five groups: two groups each in two of the municipalities, and one group in the third municipality. Considering heterogeneity, each group consisted of participants with different professional backgrounds, ages and work experience, and which represented both care settings. To ensure homogeneity, all participants had experience in long-term care, and within each group, participants worked in the same municipality and some of the members worked together. In order to achieve a group dynamic that could generate data, considerations were made to balance a feeling of security among participants and variation in experiences (Kitzinger, 1994). An overview of the focus groups is shown in Table 1.

**Table 1.** Overview of participants in the focus groups.

Focus group	Working in nursing homes (*N*)	Working in home nursing care/assisted living facilities (*N*)	Participants in total
Group 1		4	4
Group 2		6	6
Group 3	6		6
Group 4	5		5
Group 5	3	3	6

In total, 27 women participated, comprising nine registered nurses, 14 auxiliary nurses, three assistant nurses and one nursing student. We have chosen to refer to all participants as ‘nurses’ in order to conceal identities. The participants were aged 22 to 62 (median age of 46) and their nursing experience ranged from 2 to 30 years (median of 18 years). Ten of the participants worked full-time and the rest worked part-time. No participants worked less than 50% of a full-time position (median of 75%). All participants were ethnic Norwegians.

### Data Collection

Data collection was carried out by Siri Andreassen Devik and a research assistant from May 2018 to March 2019 and took place in conference rooms at nursing homes and in home nursing premises. Siri Andreassen Devik moderated the focus groups. She is a geriatric nurse with extensive clinical experience in long-term care, in addition to being an experienced researcher. The research assistant who observed and assisted in the interviews is also an experienced nurse, with a master’s degree in clinical nursing. The interviews were conducted in a manner that ensured a safe environment where the participants felt free to express their opinions. A semi-structured interview guide was developed to ensure that the groups had focused discussions about the topic (Krueger & Casey, 2000). The interview guide was based on a review of previous research and on the purpose of the study and reflected the three aspects we aimed to explore: perceptions, experiences and management of ethical challenges. Initially, the nurses were asked to describe their work environment – how it was organized and what a typical day looked like, and what made a day good or bad. Next, they were asked to talk about what they regarded as ethically challenging in their work. They were encouraged to tell stories or give examples of the difficulties and elaborate on how they experienced them and were affected by them. Finally, they were asked to exemplify how they dealt with such situations and elaborate on what they thought might be helpful when experiencing ethical challenges.

The interviews lasted 80–110 minutes and were audiotaped and transcribed verbatim by Hilde Munkeby and Siri Andreassen Devik. The audio recording failed in one interview (Group 2), but this was discovered immediately after the interview was completed and a script reproducing the interview was jointly written by the interviewer and the assistant. Data from Group 2 is included as part of the overall data, but no direct quotes are given from this interview. The number of focus groups (five) was determined in advance and was agreed with the municipalities based on the resources they could use in this project. We did not use an iterative approach when collecting the data. All data was collected before the analysis began.

### Analysis

The interview texts were analysed using qualitative content analysis (Graneheim & Lundman, 2004). In the search for content that could address the study’s purpose, our approach was inductive and text-driven. Patterns were identified by sorting the content based on similarities and differences (Graneheim et al., 2017). The interviews were first read in their entirety to get an overall impression. Each interview was treated as a unit of analysis.

Meaningful units in the text were identified and condensed into shorter descriptions. The condensed descriptions were reflected upon, interpreted and abstracted into subthemes and themes that described their underlying meanings (Graneheim et al., 2017). Our intention was to look for latent content – that is, not only what the participants said but also what they talked about. The process was not linear, but a back-and-forth movement between the whole and the parts of the text which involved a de-contextualization as well as a re-contextualization (Graneheim et al., 2017). Finally, we discovered a recurring content across the themes and sub-themes that emerged as a main theme: ethics between the lines. Hilde Munkeby conducted an initial analysis, which was discussed and revised with Siri Andreassen Devik, before all the authors discussed and came to an agreement on the final interpretation. All authors are nurses with clinical experience in long-term care, except the Grethe Bratberg who mainly has nursing experience in hospitals.

### Ethics

The Norwegian Centre for Research Data approved the handling of personal data (project no. 571,318). The participants received both oral and written information about the study purpose and procedure and gave voluntarily signed consent before the interviews were conducted. Prior to the focus group interviews, participants were reminded of their duty of confidentiality toward patients. They were encouraged to avoid detailed descriptions or information that could make patients identifiable when giving examples from their own work.

### Findings

The analysis resulted in one main theme, three themes and six subthemes. An overview of the findings is shown in Table 2, and an example of the analysis is shown in Table 3.

**Table 2.** Overview of themes and subthemes.

Main theme	*Ethics between the lines*		
Theme	*Striving to do good*	*Failing and being let down*	*Getting rid of frustrations and learning from experiences*
Subtheme	Striving to define good and what is good for whom	Failing the patient	Needing to vent frustrations
	Striving to achieve the good	Being let down	Learning through reflection on a subject

**Table 3.** Example of analysis.

Meaning unit	Condensed meaning unit	Subtheme	Theme
It hurts a little when the patient is not completely happy and says – ‘but you decide anyway…’	Difficult to feel that one decides over the head of the patient	Striving to define good and what is good for whom	*Striving to do good*
One week, there were three patients on death row, and no one should have to die alone … but no extra staff was hired. Even if we tell the management, the answer is that there is no one to hire	We were not enough personnel at work to take care of the dying, but received no help even though we requested	Being let down	*Failing and being let down*
Sometimes I have to call one of my colleagues in the evening at home…, if special things have happened at work I need to talk to someone	I need to call my colleague in the evening to talk about special incidents at work	Needing to vent frustrations	*Getting rid of frustrations and learning form experiences*

#### Ethics between the lines

*Ethics between the lines* was found as the main theme in our analysis. When the nurses talked about what they found ethically difficult, and how they were influenced and coped, the ethical dimension seemed both implicit and unclear – it was somewhat *between the lines*. Ethical challenges seemed difficult to verbalize, agree on and learn from. The nurses used their everyday language and let the examples of difficult situations speak for themselves. Sentences were often not concluded with anything other than an outburst of emotion that presupposed the audience’s understanding of the meaning. The nurses rarely used value-laden terms or concepts related to ethical theory. However, ethics materialized in the practical situations discussed and manifested most clearly in the emotions that arose. The main theme was found as a common thread throughout the three themes; *Striving to do good*; *Failing and being let down* and *Getting rid of frustrations and learning from experiences*, which the analysis resulted in.

### Theme 1: Striving to do Good

The nurses’ perception of ethical difficulties became clear in their stories about challenges in doing good for the patient. The struggles were mainly expressed by the emotions they created and often with reference to the opposite of what the nurses perceived as good. On one hand, there were difficulties in defining what good means, and what is good for whom in each situation. On the other hand, there was ambiguity about which actions could be considered good or viewed as the right actions to achieve good.

#### Striving to define good and what is good for whom

When talking about ethical challenges, participants gave rich descriptions of many troubled situations in their daily work – which they frequently referred to as frustrations. The situations could involve a variety of issues, such as everyday communication with individual patients, prioritization of resources and needs in care situations, interactions with relatives or colleagues and care at the end of life. One nurse summarized the experience by saying: ‘*To me, the ethical difficulty is the feeling of doing something wrong’* (Group 3). A characteristic of such descriptions was an absence of complete agreement on why things ‘felt wrong’ or what good was and for whom. For example, there could be different opinions about vital treatment versus a dignified life and respect for death. One nurse said:

*My impression is that recent graduates have a strong focus on treatment and commitment to saving lives … but what about the patient’s quality of life? I agree that we should actively relieve suffering but if the treatment becomes limitless … there is not much dignity in getting a fist to the chest if resuscitation is done at the end of life. (Group 1).*

The patient’s well-being was an undoubted goal for all the nurses, and a common ideal was that care should be meaningful and make a positive difference in the patient’s everyday life. However, challenges arose when the nurses understood that the patients' experience of good did not coincide with their own. One of the nurses spoke about an elderly nursing home resident who refused to receive help with personal hygiene, adjusting the circadian rhythm or reducing alcohol intake. The resident had no cognitive impairment and did not want interference in his lifestyle. In another case, a nurse described the situation of a person who had dementia and did not want to change pants and diapers that were soaking wet with urine. The nurse said: ‘*Then you come to the ethical – is it ethically correct to let the patient go into the living room where the other patients are and make it possible for them to see that he is wet with urine?’* (Group 3).

The challenge of defining the good required nurses to be able to distinguish between various parties` perspectives. It was not just a matter of the nurses having different opinions, or that the views of the patient and the nurse differed. Sometimes the opinions of relatives and the patient also did not match:

*Sometimes I wonder whose needs we are expected to satisfy. Is it our own, the patient’s or relatives'? It is difficult to relate to relatives' preferences that the patient should always be clean and pretty in his clothes or that he eats and drinks well at all times* … *even if it almost requires that we push or force him.* (Group 3).

Practical aspects of the work were also something that influenced the perception of what was good. Being very busy was a theme in all the groups, and the nurses perceived clear expectations of efficiency on the part of the employer. Balancing relatives' perceptions of good care and their own perceptions of realistic and good care, and taking into account the patient’s situation and conditions in the ward was difficult. One of the nurses said: ‘*Sometimes it is so busy that we are almost forced to feed several patients at the same time* … *from the outside it will look like we are just stuffing the food into the patients’* (Group 3).

#### Striving to achieve the good

Perceptions of ethical challenges were also marked by uncertainty and disagreement about how to achieve the good and what actions should be taken. Typically, uncertainty arose in situations when the nurses had to prioritize between different care needs and between different patients. Such situations felt bad because they resulted in some patients being ignored and some problems taking precedence over others. One nurse described this as a daily occurrence: ‘*Patients often go after us and shout at us, but you just have to walk past them because they have already received their share of help. Then you must continue with those patients who never demand anything’* (Group 3).

Difficult choices either had to be made by nurses during the actual situation or had already been made by others in advance. The latter circumstance was exemplified by the formal decisions about home nursing care that municipal caseworkers made. The nurses generally accepted formal decisions as a way to set care limits or encourage the patient’s self-care, but they also had criticisms and made objections. Compliance with formal care decisions thus varied depending on how well the decisions corresponded to what the nurses considered to be the patient’s needs and the right prioritization of needs. One of the nurses stated:

*Many of us probably perform tasks strictly according to the decision, but it is difficult not to light up the heater when the patient is sitting there and freezing … I think it is bad if we are not allowed to follow our own conscience* (Group 1).

Some of the most demanding situations involved the use of coercion. Even if the nurses held on to the idea that the end justifies the means, they could doubt whether coercion really achieved good for the patient. One of the examples was a situation where three or four nurses were needed to carry out morning care for a patient:

*I think it’s the worst thing I’ve been through since I started working in the early ‘80s. We really have to hold the patient tight … it feels like abuse. The alternative is that the patient is not cared for … it does not feel good for us either, but I wonder if it could have been done differently* (Group 3).

### Theme 2: Failing and Being Let Down

Experiencing ethical challenges often led to a feeling of failing the patient. However, the nurses also felt that they themselves were let down and placed in positions where they could not act in accordance with what was good.

#### Failing the patient

To be unable to stand up for patients and provide the care that they believed was necessary gave the nurses a bad conscience and a sense of guilt. One nurse said: ‘*What do we feel? A bad conscience, because you are not able to do your job, they [the patients] don’t get the care they need, they are not well at all’* (Group 5). This involved many situations, for example, not being able to protect the patient from unwanted exposure to others (patients and relatives) or failing to ensure patients’ sufficient treatment and care. One of the nurses cried when she reported:

*When we finally persuaded the doctor to look at her, she [the patient] was hospitalized. After a day or two she died … when the family came for her belongings I didn’t even manage to look at them, I just cried* (Group 1).

To participate in coercion represented a clear experience of failure, but witnessing colleagues’ rude behaviour or communication without intervening also felt like a betrayal. Lack of courage to confront each other’s behaviour was one explanation, and in such situations, patients’ needs and rights seemed subordinated to preserving a good relationship with colleagues. One nurse recounted how a colleague confided that she had witnessed another nurse hit a patient in the face. The nurse reproduced the conversation: ‘*I was completely speechless when she told me this, but after a while I asked: “What did you do or say then?” “Nothing”, she said. “I didn’t dare’*”’ (Group 1).

### Being Let Down

The nurses struggled not only with feelings of failing the patients, but also with feelings of being offended or let down by patients or their next of kin. They felt that their self-confidence was affected when they were beaten, spat on or bullied by patients. One nurse said: ‘*You go from one room to another and just hear negativity, how you look and don’t look … then you just get … “God knows what!’*”’ (Group 1). In some situations, nurses’ motives were also questioned. A nurse explained:

*We have some relatives at our ward who say that we treat patients worse than animals … it’s not good to hear, it makes me sad, because it’s not what we do. It’s not the way it is … that we prioritise those who shout the loudest* (Group 3).

The nurses felt that they were largely held responsible for the system and at the same time were prevented from explaining why the routines or resources were a certain way.

A feeling of being let down by their employer or organization was clearly expressed and was, in the nurses’ opinions, part of the ethical aspect as well. One nurse said, ‘*the ethics also involve what happens to us … what we have to live with’* (Group 1). There were large variations in how the nurses perceived their general workload. Some participants described themselves as not only being exhausted due to lack of time and personnel but also trapped by the system, and suffering from lack of leadership involvement and support. The nurses gave examples of how attempts to describe their situation to managers were unsuccessful. The answer was that they could not expect more resources due to the municipality’s finances. Others did not complain about time limits and had leaders who were highly involved in their daily work: ‘*The barrier to accessing the leader is low, and she will always listen and guide us’* (Group 4).

### Theme 3: Getting Rid of Frustrations and Learning from Experiences

In order to cope with ethical challenges, the nurses identified that they needed to vent their frustrations, as well as reflect on and learn from the situations they were involved in.

#### Needing to vent frustrations

The nurses said that they often eased the pressure by sharing their frustrations with colleagues. Talking to colleagues felt both safe and relieving. One nurse explained: ‘*It feels like therapy. It is not only me being fragile … others feel the same, it’s normal’* (Group 3). They preferred to reflect with colleagues with whom they either worked the same shift or felt a close relationship. Several nurses gave examples of contacting colleagues in their spare time if they needed to talk about incidents at work.

Some of the nurses approached their leader, but felt that the leader was rarely there when they needed them, and that the leader’s approach to challenges was different from their own: ‘*They [the leader] take a more theoretical approach to the situation, while we take the emotional side of it’* (Group 5).

The need to reflect could arise at any time during the day. As one nurse said: ‘*Sometimes we have to go into the storage room, just to get together for a few minutes and let each other vent’* (Group 1). The trigger for a need to debrief could be of a more or less dramatic nature. While some nurses had the opportunity to spend working time reflecting and talking about various situations, others had almost no time except for the report time between shifts.

#### Learning through reflection on a subject

Most of the nurses had experience with ethical reflection groups. At some workplaces, the reflection was carried out fairly regularly, while some nurses had only experienced it for a short project period. The experiences varied. Some nurses found the reflection useful and something that increased their ethical awareness, but others found it artificial and distant from daily practice. Several nurses agreed that ethical reflection could become somewhat empty and meaningless if they could not relate it to anything. One nurse explained: ‘*Just the fact that it was called ethical reflection, it would be better if they didn’t call it anything and just addressed a topic … Ethical reflection, it sounds a bit strange’* (Group 5). Another nurse added:

*I think it’s a bit artificial when choosing a reflection card to have something to discuss. You may learn a method, but it is much more useful if you can present an actual situation, an ethical challenge that you have experienced and can get something out of (Group 5).*

Other nurses had the opposite experience of using cards as a starting point for reflection and found it useful for getting on the trail of ethics. One nurse said: ‘*Not everyone understands what ethics is all about. Then the card can be eye-opening … at least I know we started talking about things that surprised me’* (Group 1).

Overall, it seemed that ethical reflection needed a subject with practical significance for the nurses to see the benefit of the exercise. Reflecting on specific cases gave the nurses an opportunity to learn from each other. One of the nurses explained:

*You listen to what others say and think about it … and then you can discover other ways of thinking and doing. You also get feedback and in some way a sense of security. It becomes less dangerous and difficult. If we dare to open up, it does something for the whole work environment, I think* (Group 1).

Those who experienced the reflection as disarming also felt that it gave room to challenge and learn from each other. As one nurse said: ‘*We can also stop each other, we can say: “Dear, what did you think when you did that?*”’ (Group 4).

## Discussion

The findings of this study contribute to a bottom-up perspective on ethical difficulties, as nurses in long-term care convey in their own words and feelings. The nurses in this study experienced ethical challenges when trying to do good, including deciding what was good in the actual situation, what was good for whom and how to best achieve the good. These ambiguities created emotional unease and involved choices that could create frustration, pain and feelings of betrayal. It felt painful to fail patients, but the nurses also felt let down themselves when put in situations where they could not act in accordance with their own conscience. The nurses perceived that working conditions often made it impossible to fulfil the good, but were not something they could explicitly use as an explanation. The nurses sought support from each other and needed to comfort themselves and to understand and learn from the challenges they faced. However, the necessary attention from leaders, legitimate room for reflection and a familiar language were not always available.

Our overall impression when listening and talking to the nurses was that ethics was implicit in their communication and rarely expressed directly with concepts related to ethical theory. fleeting in their communication. The nurses also admitted that they struggled a bit with the concept of ethics and felt that ethical reflection could be both distant and artificial. Still, their ethical awareness and reflexivity were clearly present and vivid in the practical examples they gave.

Nurses’ sense of alienation with respect to moral theories is nothing new. According to Peter and Liaschenko (2003), the dominant bioethical theory does not anchor nurses’ identities, responsibilities or the moral experiences of their work. The consequence of such alienation might be that what nurses consider ethical problems are redefined as merely practical problems, which in turn contributes to making their concerns invisible (Peter & Liaschenko, 2003).

Ethical difficulties related to lack of resources or restrictive work instructions were concerns that the nurses in this study found especially difficult to raise. The nurses were clear that ethics also applied to how they felt and what happened to them. However, problems concerning resources and routines are often seen as practical and non-ethical (Haahr et al., 2020), and to the extent that they lead to ethical difficulties, the responsibility may be placed on the individual nurse (Devik et al., 2020). In encounters with patients and relatives, the nurses are thus left with both their own and the organisation’s responsibilities. The nurses gave examples of situations where they could not defend themselves against accusations from relatives or avoid giving the impression of being immoral. Consideration for the patient, and the responsibility to do good and not fail the patient, weighed heavily in such examples. Meanwhile, the feeling of being let down themselves had to be suppressed. The emphasis on the nurse-patient relationship is both important in care and is especially apparent in professional nursing ethics literature. However, the relationship is not in a vacuum and happens in a collaborative and complex healthcare environment with agendas that nurses often have little opportunity to influence. Liaschenko and Peter (2004) argue that professional ethics do not sufficiently take into account this interaction or recognize the complexity of what nurses are required to do to achieve good for the patient.

To shed light on the phenomenon that ethics may be perceived as invisible, we find the thoughts of Margaret Urban Walker (1998) highly relevant. Walker argues that morality is ‘a socially embodied medium for mutual understanding and negotiation between people over their responsibility for things open to human care and response’ (p. 9). The responsibility for doing good was deeply felt and shared among the nurses in this study. However, they struggled to define the content of the good, for whom that content would be good and in what ways good could be achieved. The situations that were perceived as ethically challenging related to lack of time, personnel and sufficient medical and management support; collaboration problems with patients, relatives or colleagues and challenges in meeting the various needs of different patient groups. Ethical issues concerned communication, decision-making and prioritization in everyday situations as well as end of life care, in line with previous research (Bollig et al., 2015; Heggestad et al., 2020; Preshaw et al., 2016). However, the nurses’ morality was expressed first and foremost through the emotions that arose in specific situations with patients and not via theoretical concepts (cf. Walker, 1998).

Although the nurses rarely referred to ethical theory, their examples showed that different opinions were negotiated and contested. The nurses in our study felt a strong need to discuss their difficulties with their colleagues. Having an outlet for emotions and sharing and discussing experiences with others were both important. Walker (1998) assumes that morality is a collaborative practice of responsibilities. This implies a need to reach common understandings, to negotiate with others to determine the scope of the agency, confirm identity and values and make actors accountable to each other (Walker, 1998).

Through reflection, these understandings and responsibilities can be critically examined to ensure that they are acceptable and coherent to those who hold them. All the nurses in our study had participated in ethics reflection groups. The reflection was experienced as useful in terms of enhanced ethical awareness and improved collaboration in the healthcare team, which is in line with earlier research (Lillemoen & Pedersen, 2013; Magelssen et al., 2018; Söderhamn et al., 2015). The premise seemed to be that the reflection was based on a concrete subject. Some methods of ethical reflection are based on what is morally right according to policies and laws (Schuchter & Heller, 2018), while other methods focus on how participants perceive and construct moral rightness where legal or ethical principles might be a part of the dialogue, but not necessarily (Molewijk et al., 2011). The latter approach, known as moral case deliberation, acknowledges emotions and can promote a better understanding of oneself and give emotional support like comfort and enhanced resilience (Molewijk et al., 2011; Spronk et al., 2020). This accords with our findings. Working with emotions is part of such reflection, but it also includes helping to identify and articulate the nurses’ own values and responsibilities in their work (Verkerk & Lindeman, 2009). Participating in a reflection group can in this sense make tacit and unclear ethical concerns visible and verbal, both in the situation and as normative arguments.

Moreover, our findings showed that group reflection could contribute to an environment that felt sufficiently safe to embolden nurses to confront each other and adjust behaviour. The reflection group thus becomes an arena for morality as a social achievement (Walker, 1998) that takes place between people when they are held accountable to each other and themselves for their obligations. In reflection groups, the nurses had the opportunity to explore and clarify their identities, relationships and responsibilities, but it seemed to us that one party was mainly missing from the table – namely, the leader. Perhaps it would be helpful for both managers and nurses to participate in ethical reflection together. We have not found information on the extent to which this actually happens, but we believe that this is an important issue to pursue in future research. Leaders’ attention and support is undoubtedly important, according to our findings and those of others (Lillemoen & Pedersen, 2015; Makaroff et al., 2014).

Nurses in long-term care need dedicated spaces to develop their ethical competence. The lack of useful concepts in communication about ethics may also have implications for education. Concepts used in ethics education must be clear and communicated so that content and meaning have practical relevance and can be used by future nurses. There is a need to strengthen cooperation between long-term care and education in order to bring practical situations to education and theory into practice. Nurses also need leaders who recognize that resource constraints and organizational conditions affect patient interactions that the individual nurse faces. This implies that leaders must recognize the connection between nurses' work situation and the moral anguish that is created. Facilitating ethical reflection is an important measure, which may also require leaders to actually participate.

### Strengths and Limitations

The participants in this study represented extensive professional experience and a mix of nursing skills that is typical in this setting. The groups were composed to facilitate diversity and capture opinions from nurses with different backgrounds. The participants also represented different workplaces, where some knew each other well while others were unknown to each other. These variations may have influenced the dialogue and interaction in the group. An alternative analysis strategy, with a focus on interaction rather than content, could have revealed possible power relations between the participants (Smithson, 2000). In addition, we cannot ignore the fact that some participants may have found it difficult to talk about ethics in the hearing of others. Some participants may have felt uncomfortable sharing some details.

Invitations went out to both male and female nurses, but the sample in this study only represents the perspective of women. This may constitute a limitation, but also reflects the predominance of women working in this setting (Fagertun & Tingvold, 2018). The number of focus groups (five) was determined in advance and agreed with the municipalities based on the resources they could use in this project. Therefore, an iterative approach was not used in the process of collecting and analysing the data. However, we were concerned with depth of content in order to be able to identify potential latent meaning rather than saturation in the form of repetition or a lack of new themes. Questions about how saturation can be achieved will vary depending on the design of the study as well as practical conditions. We placed emphasis on describing well who the participants were and what data they provided (Coenen et al., 2012; Sebele-Mpofu, 2020). Interpretations and findings were continuously discussed in the research team, and agreement on the final understanding was reached.

One interview was not audiotaped, but a script written afterwards was used in the analysis. According to Rutakumwa et al. (2020), the data quality of audio-recorded transcripts and interview scripts is comparable in the detail captured. Our findings correspond with previous research in the field and transferability is strengthened by the use of established theory.

## Conclusion

Ethical challenges were experienced in trying to do good, including deciding what was good in the actual situation, for whom it was good and how best to achieve the good. Concerns related to organizational limitations often led to feelings of failing the patient, and the nurses also felt let down themselves. Organizational barriers are not just a practical issue for nurses – they also cause ethical difficulties. If nurses' ethical concerns are disregarded or left to be dealt with by the individual nurse, quality of care as well as sustainable staffing in this sector may be threatened.

Ethical reflection that takes seriously the challenges that nurses consider ethically difficult and that enables the understanding of specific situations is a necessity. Nurses in long-term care need dedicated spaces to develop their ethical competence, and they need leaders who acknowledge that the responsibility for good care is a shared one.

## Supplemental Material

sj-pdf-1-gqn-10.1177_23333936211060036 – Supplemental Material for ‘Ethics Between the Lines’ – Nurses’ Experiences of Ethical Challenges in Long-Term Care Click here for additional data file. Supplemental Material, sj-pdf-1-gqn-10.1177_23333936211060036 for ‘Ethics Between the Lines’ – Nurses’ Experiences of Ethical Challenges in Long-Term Care by Hilde Munkeby, Aud Moe, Grete Bratberg and Siri A. Devik in Global Qualitative Nursing Research
