# Supplementary material for: ‘Ethics Between the Lines’ – Nurses’ Experiences of Ethical Challenges in Long-Term Care
Source: Glob Qual Nurs Res. 2022 Jan 5;8:23333936211060036. doi: 10.1177/23333936211060036 (PMC8738871; doi:10.1177/23333936211060036)
Supplement: sj-pdf-1-gqn-10.1177_23333936211060036 – Supplemental Material for ‘Ethics Between the Lines’ – Nurses’ Experiences of Ethical Challenges in Long-Term Care [file sj-pdf-1-gqn-10.1177_23333936211060036.pdf]

Interview guide

Can you first tell us a little about your everyday work?

- Which patients do you take care of? How is the work organized?
- What is a normal working day looking like?
- What makes a day good or bad?

What do you perceive as ethically challenging in your everyday work?

- Please, give examples or tell about events

How are you affected by such experiences?

- How do you feel?
- How do you react and act?

How do you cope with being in ethically difficult situations?

- What do you do?
- What have you experienced?

What do you find useful when you experience ethically difficult situations

- Is there anything you can do yourself
- Is there anything your employer can do?

The questions were repeated and directed at the participants so that everyone could respond. Follow-up questions were asked when someone gave examples such as - what do you think when you hear this? - is there anything you recognize? - or do you have other experiences? Can you elaborate on this?
